# Genetic analysis of growth parameters and survival potential of Jamunapari goats in semiarid tropics

**DOI:** 10.1016/j.smallrumres.2018.04.002

**Published:** 2018-08

**Authors:** P.K. Rout, O. Matika, R. Kaushik, M.S. Dige, G. Dass, M.K. Singh, S. Bhusan

**Affiliations:** a Genetics and Breeding Division, ICAR-Central Institute for Research on Goats Makhdoom, Farah, Mathura 281122 Uttar Pradesh, India; b Genetics and Genomics Division, The Roslin Institute and R (D) SVS, University of Edinburgh, UK

**Keywords:** Body growth, Average daily gain, Genetic trend, Jamunapari goats

## Abstract

Goats are adaptable to varied farming environments and contribute significantly towards sustainable livestock production and food security. Selective breeding of goats for improved performance is an important component of sustainable production. The objectives of the present study were to determine the most appropriate models of analysis for growth traits, to estimate genetic parameters, survival potential of kids from birth to 12 months of age and to estimate genetic trends for growth traits of Jamunapari kids at different ages. Genetic parameter estimates were obtained from 6590 records generated between 1982 and 2012 from 5922 animals with a pedigree covering over 13 generations. The most parsimonious model for early growth traits included permanent environmental effects due to the dam (PE) and litter effects. Similarly, the most appropriate model for early average daily gain (ADG) between birth and 3 or 6 months also included PE and litter effects. The estimates of heritability for survival from birth to 12 months of age ranged from 0.10 to 0.43. The estimates of heritability for ADG varied from 0.04 to 0.41. In general, higher estimates of heritability were observed when a sire model was fitted in the random effect. There was no genetic variation observed for survival between birth and 3 months of age. However, heritability estimates of between 0.18 and 0.39 were observed for survivability during post-weaning period to 12 months of age. The genetic trend at 9 months of age and 12 months of age was 0.144 kg 0.189 kg per year respectively. The genetic trend at all the ages was positive during the study period.

## Highlights

• The estimates of heritability for body weight from birth to 12 months age ranged from 0.10 to 0.43.
• The estimates of heritability for average daily gain (ADG) during various growth phase varied from 0.04 to 0.41.
• The heritability estimates of survival potential for post-weaning period to 12 months of age varied from 0.18 to 0.39.
• The genetic trend of body growth traits at 9 months of age and 12 months of age was positive.

## 1 Introduction

The rapidly growing livestock market and the opportunity for global trade offer the potential to increase the income of livestock farmers. Moreover, the global preference for animal protein consumption has increased over last decade. Since goats (*Capra hir*cus) can utilize fibrous plant materials to produce meat, which offers a reliable source of animal protein in developing countries, their abundance may have led to an increasing preference for goat meat in developed countries (Devendra, 2010; Webb et al., 2005). Goat meat (chevon) is the most preferred meat in India and the domestic demand outstrips its supply (Sen et al., 2004). More importantly, goats produce meat and milk in different types of agro climatic zone and supplement the nutritional requirement of human-beings in several disadvantaged geographical locations. Goats are adaptable and resilient animals for sustainable livestock production and have the potential to fulfill the protein requirement of growing population, with most of the approximately 1 billion world goat population, 56 and 30% located in Asia and Africa respectively (FAO, 2015). Moreover the carcass fat content of goat meat is low and virtually all the parts of carcass are consumed (Webb, 2014). Therefore, it is necessary to optimize productivity of goat breeds with naturally available feed resources and goat producers have to adopt new technologies and better feeding practices to improve the performance and yield of goats (Webb, 2014). Body weights and average daily gains are important selection traits for improving production performance by selective breeding. Selection is primarily based on body weight between 6 and 12 months of age, with increasing body weight considered as important breeding goal. However, in practice, the most appropriate genetic model should be considered when breeding for any trait that is evaluated. Goats are not generally well studied, resulting in limited genetic parameter estimates available on growth, milk and reproduction traits (Bosso et al., 2007; Boujenane and El-Hazzab, 2008; Zhang et al., 2008; Gowane et al., 2011; Rashidi et al., 2011). Genetic parameters on growth traits have been reported in different breeds and locations (Snyman and Olivier, 1996; Roy et al., 2008; Hermiz et al., 2009; Thiruvenkadan et al., 2009; Alade et al., 2010; Ekambaram et al., 2010; Roy et al., 2011). The heritability of growth traits at different ages varied from 0.06 to 0.88 in different breeds (Mukundan and Bhat, 1978; Madeli and Patro, 1984; Jin and Zhang, 1995; Bishop and Russel, 1996; Snyman and Olivier, 1996; Schoeman et al., 1997; Rashidi et al., 2000; Rashidi et al., 2008; Roy et al., 2008; Hermiz et al., 2009; Thiruvenkadan et al., 2009; Ekambaram et al., 2010; Faruque et al., 2010; Roy et al., 2011). There are few reports on genetic parameter estimation of average daily gain between different growth stages and survival potential. Moreover genetic trend of growth traits has not been reported in goat breeds in different regions. Therefore, the objectives of the present study were (a) to determine the most appropriate models of analysis for body weight and average daily gain (ADG) of Jamunapari kids at different ages; (b) to estimate genetic parameters of these traits; (c) to estimate the genetic parameter of survival potential of kids up to 12 months of age; and (d) to evaluate the genetic trends in body weight during the last 31 years of selective breeding.

## 2 Material and methods

### 2.1 Animals

The Jamunapari goats were introduced to the study area (CIRG) from their natural habitat, the Chakarnagar area of Etawah district of Uttar Pradesh, which is situated 150 km from the Central Institute for Research on Goats (CIRG) in Mathura, India. The study area has semi-arid climate and an average annual rainfall of about 375 mm which is scattered during the months of June to September. The soils are sandy with natural pasture and bush as the main vegetation type. The pastures are mainly *Cenchrus ciliaris* and *C. setigerus*, along with native annual and perennial flora. The temperature varies from 4.0 °C to 24.3 °C during winter and 27.5 °C to 42.4 °C during summer.

The Jamunapari goat is a milk-producing breed with average body weight of 28.0 kg at 12 months of age with 1.5 kidding rate. It has a majestic white colour coat with long pendulous ear, Roman nose and has *trans*-boundary distribution (Rout et al., 2000). The breed has been used extensively for upgrading local breeds in neighboring Southeast Asian countries and is an ancestor to the Anglo-Nubian goat (Rout et al., 2004).

The goats were maintained under a semi-intensive system of management with 6–7 h of grazing and stall feeding with seasonally available green fodder, supplemented with concentrate mixtures (70% maize and 10–15% oil cake, 10–15% bran and mineral mixture) depending upon the status and age category of the animals. Generally, animals were housed separately according to their ages, sex, physiological status and health status. Controlled breeding was practiced with the does being bred during May to June and October to November followed by kidding in the months of October to November and March to April, respectively. Does were exposed to the bucks twice at each oestrus. At kidding, each kid was assigned an identification number by ear tattooing and date of birth, sex, birth type and live body weights were recorded. Kids were stall-fed up to weaning at 3 months of age, and then allowed to graze nearby areas for very short periods for up to 6 months of age. The preventive health care measures were regular vaccination of pestis-de-petitis (PPR), Foot and Mouth disease (FMD), and Enterotoxaemia (ET) of the flock. Targeted deworming for the control of gastrointestinal nematodes was carried out during the pre-monsoon season (May to June) and in the post-monsoon season (September to October). Animals were regularly dipped to control ecto-parasites in the flock.

### 2.2 Traits analysed

Growth data and pedigree were available from 1982 to 2012 on Jamunapari flocks maintained at the ICAR-Central Institute for Research on Goats (CIRG). The traits analysed were those for body weights at: (a) birth (BWT), 3 months (M3WT), 6 month (M6WT), 9 month (M9WT) and 12 months (M12WT) of age; (b) average daily body weight gain (ADG) between birth to 12 months of age within the following periods: birth to 3 months (0–3 M), 3–6 months (3–6 M), 6–12 months (6–12 M), birth to 6 months (0–6 M), birth to 9 months (0–9 M), birth to 12 months (0–12 M), 3–9 months (3–9 M), 3–12 months (3–12 M); and (c) survivability of kids from birth to 12 months of age (whether a kid born alive was still alive at weaning, 3 month, 6 month, 9 month and 12 month of age). Pedigree records on 5922 animals over 13 generations were used for genetic parameter analysis (Table 1).

**Table 1:** Summary of pedigree data structures and number of records.

Source	Numbers
Phenotype records	6590
Pedigree records	5922
Sires	292
Sire of sires	125
Dam of sires	196
Dams	1819
Sire of dams	207
Dam of dams	784
Generations	13

### 2.3 Data analysis

Initially data were explored for summary statistics and normality using SAS (SAS, 2013). The models fitted accounted for environmental effects for parity (with all parities above 6 fitted as one class), year (1982–2012), season (autumn and spring) and birth type (TOB, singles and multiple with 2 plus treated as one class). Random effects for animal, sire, maternal (m) and permanent environmental (PE) effects due to the dam were fitted. Estimates of variance and co-variance components were obtained using the *ASreml* program (Gilmour et al., 2009), initially fitting the univariate models. The following models were fitted:

(1) *Model 1: y* *=* *Xb* *+* *Z_a_* *+* *e*

(2) Model 2: y = Xb + Z_a_ + Z_m_ + e

(3) *Model3: y* *=* *Xb* *+* *Zs* *+* *e*

(4) *Model 4: y* *=* *Xb* *+* *Z_a_* *+* *Zpe* *+* *e*

(5) *Model 5: y* *=* *Xb* *+* *Z_a_* *+* *Zpe* *+* *Zlit + e*

(6) *Model 6: y* *=* *Xb* *+* *Zs* *+* *Zpe* *+* *Zlit* *+* *e*

Where *y* is a vector of observations on specific traits of the animal; *b* is a vector of fixed effects; *a, m, s, pe, lit* are vectors of random effects describing additive genetic, maternal additive, sire, litter and permanent environment effects due to dam; X, Z are corresponding incidence matrices relating to each effect to *y*; and *e* is the vector of residuals. Normal distributions were assumed for the random effects: direct additive ∼N (0, Aσ^2^_a)_, sire ∼N (0, Aσ^2^_sire)_, pe ∼ N (0, Iσ^2^_pe_), litter ∼N (0, Iσ^2^_lit_), e ∼ N (0, I σ^2^_e_), where A is the numerator relationship matrix, and I denotes the identity matrices of the order equal to the number of litters and records. Likelihood ratio tests (LRT) were carried out to determine the most suitable model for each trait in univariate analyses (Morrell, 1998). The test statistic was −2[lnL_(2)_-lnL_(1)_] where L_(n)_ is the likelihood of Model n. Critical values for the LRT were taken from a mixture distribution ½$\chi_{}^{2}$_(1)_ and ½$\chi_{}^{2}$_(0)_ (Self and Liang, 1987).

Breeding values of individual animals were estimated with ASReml. In order to estimate the genetic trends, means of estimated breeding values of the kids within year of birth were calculated. Genetic trends were obtained by regression means of estimated breeding values on year of birth for each trait.

## 3 Results

### 3.1 Body weights at different ages

The 6590 phenotypic records were obtained from 292 sires and 1819 dams during the 31 years. The complete summary statistics for all growth traits analysed, as well as means and coefficient of variation (CV) are presented in Table 2. There was wide range of variation in body weight gains during different growth periods over the years. The wide range of variation in body weight at different ages was observed as there was no culling practiced till 12 months of age for obtaining information on all the individuals. The least squares analysis of variance indicated that parity of dam, year, birth type and sex had significant effect (P < 0.05) on body weight at different ages. Season of birth had significant effect (P < 0.01) on body weight at 3 months of age.

**Table 2:** Summary statistics for growth traits of Jamunapari goats.

	BWT	M3WT	M6WT	M9WT	M12WT
Number of Records	5922	5359	4758	4223	3756
Mean (kg)	3.1	10.3	14.5	19.4	23.9
Standard Deviation	0.67	2.42	3.53	4.92	6.03
Standard error	0.01	0.03	0.05	0.08	0.10
Coefficient of Variation	21.3	23.5	24.5	25.4	25.2
Range (Minimum-Maximum) Kg	0.8–5.6	3.0–21.0	4.0–28.6	6.0–41.0	8.5–48.2

Where BWT ∼ birth weight; M3WT∼ 3 months weight; M6WT∼ 6 months weight; M9WT∼ 9 months weight; M12WT∼ 9 months weight

The most “appropriate” model for growth traits was the complete model accounting for both permanent environmental effects due to the dam and the common environment for litter effects from birth to 9 months when fitting an animal model and from birth up to 12 months of age for the sire model (Supplementary Table S1).

Estimates of additive heritability increased from birth to between 6 and 9 months of age and decreased subsequently during 9 month and 12 months of age irrespective of the model fitted (Table 3). The estimates of heritability for the sire model were higher between 3 and 6 months of age. The common environment for litter effects were higher across models (0.27–0.39) during birth to 3 months of age and decreased thereafter during 6–12 months of age (0.12–0.16). The variance component due to permanent environment due to dam are generally important earlier in life at birth to 3 months of age and decreased subsequently during 6–12 months of age observed for the animal model.

**Table 3:** Genetic parameter estimates for growth traits between birth and 12 month of age fitting an animal or sire model accounting for permanent environmental effects due to dam (pe) and litter effects (lit).

Trait	BWT		M3WT		M6WT		M9WT		M12WT	
Model	Animal	Sire	Animal	Sire	Animal	Sire	Animal	Sire	Animal	Sire
σ^2^_a_	0.046		0.651	–	1.613	–	1.652	–	1.854	–
σ^2^_sire_	–	0.009	–	0.455	–	0.799	–	0.375	–	0.200
σ^2^_pe_	0.034	0.046	0.397	0.554	0.625	0.995	0.573	1.080	0.577	1.159
σ^2^_lit_	0.125	0.156	1.160	1.117	1.124	1.007	2.022	1.915	2.821	2.682
σ^2^_res_	0.119	0.142	1.815	2.072	4.991	5.764	9.085	9.940	12.204	13.093
σ^2^_pheno_	0.324	0.323	4.023	4.198	8.354	8.565	13.332	13.311	17.456	17.512
s.e.	0.007	0.69	0.09	0.110	0.193	0.222	0.311	0.307	0.425	0.430
h^2^	0.14	0.11	0.16	0.43	0.19	0.37	0.12	0.11	0.11	0.13
s.e.	0.03	0.03	0.03	0.07	0.03	0.07	0.03	0.04	0.03	0.04
pe^2^	0.11	0.14	0.10	0.13	0.07	0.12	0.04	0.08	0.03	0.07
s.e.	0.02	0.02	0.02	0.02	0.02	0.02	0.02	0.02	0.02	0.02
lit^2^	0.39	0.39	0.29	0.27	0.13	0.12	0.15	0.14	0.16	0.15
s.e.	0.02	0.02	0.02	0.02	0.03	0.03	0.03	0.03	0.03	0.03

Where σ^2^_a_ ∼ direct additive variance; σ^2^_sire_ ∼ Sire variance; σ^2^_pe_ ∼ variance of permanent environment due to the dam; σ^2^_lit_ ∼variance due to Litter effects; σ^2^_res_ ∼residual variance; σ^2^_pheno_∼ total phenotypic variance; s.e. ∼ standard error; BWT ∼ birth weight; M3WT∼ 3 months weight; M6WT∼ 6 months weight; M9WT∼ 9 months weight; M12WT∼ 12 months weight.

### 3.2 Average daily gain (ADG) during different growth phase in Jamunapari goats

The “best” model for ADG in early life (0–3 and 0–6 months of age) for both animal and sire models included both common environmental effects due to dam and litter. However, for earlier growth effects (3–6, 0–9 and 0–12 ADG), the “best” model contained both environmental (PE and litter) effects in both animal and sire model. Litter effect was significant during all other ages of ADG traits for the models fitting animal as a random effect. The full description of all models showing the higher logL is described in supplementary Table S2.

The variance components for ADG were presented in Tables 4 and 5 for the animal and sire model. The highest estimates for ADG (0.18–0.20) were observed for earlier growth rates (0–3, 0–6 months) in the animal model, otherwise all other estimates were lower (0.04–0.15) (see Table 4 for more details). The estimates of direct additive heritability in the sire model were much higher (0.41–0.47) in the earlier growth traits (0–3 and 0–6 ADG) than those observed in the animal model. In general, the reminder of estimates from the sire model were higher (0.1–0.37) than those observed in the animal model (Table 5). Higher estimates of litter effects were observed from birth to 3 months of age irrespective of the model used (0.27–0.29). However, litter effects were lower for the other ADG traits analysed (0.11–0.19) across the two models assessed (Table 4, Table 5). The estimates of environmental effects due to dam were relatively low (0.01–0.11) across the models (Table 4, Table 5).

**Table 4:** Genetic parameter of average daily gain on growth traits fitting animal model, permanent environmental effects due to the dam (pe) and litter effects (lit).

	ADG								
Months (M)	Birth – 3M	3–6M	3–9M	3–12M	Birth – 6M	6–9M	Birth – 9M	9–12M	Birth – 12 M
model	pe_lit	pe_lit	lit	lit	pe_lit	lit	pe_lit	lit	pe_lit
σ^2^_a_	0.0079	0.0072	0.0023	0.0017	0.0047	0.0020	0.0022	0.0025	0.0014
σ^2^_pe_	0.0030	0.0003	–	–	0.0013	–	0.0005	–	0.0003
σ^2^_lit_	0.0130	0.0083	0.0044	0.0024	0.0031	0.0097	0.0025	0.0094	0.0019
σ^2^_res_	0.0204	0.0328	0.0199	0.0136	0.0147	0.0437	0.0119	0.0363	0.0090
σ^2^_pheno_	0.0444	0.0485	0.0266	0.0177	0.0239	0.0555	0.0171	0.0481	0.0125
s.e.	0.0010	0.0011	0.0006	0.0004	0.0006	0.0012	0.0004	0.0011	0.0003
h^2^	0.18	0.15	0.09	0.10	0.20	0.04	0.13	0.05	0.11
s.e.	0.03	0.03	0.02	0.02	0.03	0.02	0.03	0.02	0.03
pe^2^	0.07	0.01	–	–	0.06	–	0.03	–	0.02
s.e.	0.02	0.02	–	–	0.02	–	0.02	–	0.02
lit^2^	0.29	0.17	0.16	0.14	0.13	0.18	0.15	0.19	0.15
s.e.	0.02	0.03	0.03	0.03	0.03	0.03	0.03	0.03	0.03

Where σ^2^_a_ ∼ direct additive variance, σ^2^_pe_ ∼ variance of permanent environment due to the dam; σ^2^_lit_ ∼variance due to Litter effects, σ^2^_res_ ∼residual variance, σ^2^_pheno_∼ total phenotypic variance, s.e. ∼ standard error.

**Table 5:** Genetic parameter of average daily gain (ADG) on growth traits fitting sire model, permanent environmental effects due to the dam (PE) and litter effects (LIT).

	ADG								
Months(M)	Birth – 3M	3–6M	3–9M	3–12M	Birth – 6M	6–9M	Birth – 9M	9–12M	Birth –12M
model	pe_lit	pe_lit	lit	lit	pe_lit	lit	pe_lit	lit	pe_lit
σ^2^_sire_	0.0055	0.0046	0.0009	0.0010	0.0025	0.0017	0.0004	0.0021	0.0004
σ^2^_pe_	0.0050	0.0018	–	–	0.0024	–	0.0011	–	0.0007
σ^2^_lit_	0.0124	0.0072	0.0047	0.0026	0.0027	0.0100	0.0024	0.0093	0.0018
σ^2^_res_	0.0236	0.0364	0.0211	0.0143	0.0170	0.0442	0.0131	0.0372	0.0096
σ^2^_pheno_	0.0466	0.0500	0.0267	0.0180	0.0246	0.0559	0.0170	0.0487	0.0126
s.e.	0.0012	0.0013	0.0006	0.0005	0.0007	0.0013	0.0004	0.0012	0.0003
h^2^	0.47	0.37	0.14	0.23	0.41	0.12	0.10	0.17	0.14
s.e.	0.07	0.07	0.04	0.06	0.07	0.04	0.04	0.05	0.05
pe^2^	0.11	0.04	–	–	0.10	–	0.07	–	0.05
s.e.	0.02	0.01	–	–	0.02	–	0.02	–	0.02
lit^2^	0.27	0.14	0.18	0.14	0.11	0.18	0.14	0.19	0.14
s.e.	0.02	0.03	0.03	0.03	0.03	0.03	0.03	0.03	0.03

Where σ^2^_sire_ ∼ Sire variance, σ^2^_pe_ ∼ variance of permanent environment due to the dam, σ^2^_lit_ ∼variance due to Litter effects, σ^2^_res_ ∼residual variance, σ^2^_pheno_∼ total phenotypic variance, s.e. ∼ standard error

### 3.3 Survival potential

In general, the most appropriate model for survivability of kids at different ages included PE and litter effects in sire model when fitted both as linear or logit transformed (Table 6). The estimates of heritability were very low (0.0–0.03) when assessed at 3 months of age for both linear and transformed models. The heritability estimates were highest (0.39) at 9 months of age and decreased to 0.31–0.33 at 12 months of age for both analyses. Litter effects were higher (0.20–0.21) in the untransformed data at 3–6 months of age. In general, the estimates of environmental effects due to the dam were low (0.01–0.06) across all models.

**Table 6:** Survival potential of Jamunapari goats from birth to 3 (3M), 6 (6M), 9 (9M) and 12 (12M) months of age using a sire model fitting either linear or generalized mixed model (Logit transformation).

Transformation	Linear				Logit			
Trait	3M	6M	9M	12M	3M	6M	9M	12M
σ^2^_sire_	0.0006	0.0091	0.0179	0.0161	0.0000	0.1797	0.4010	0.3201
σ^2^_pe_	0.0011	0.0044	0.0073	0.0016	0.0885	0.2027	0.2256	0.0278
σ^2^_lit_	0.0163	0.0283	0.0241	0.0318	0.5754	0.3304	0.1397	0.2832
σ^2^_res_	0.0584	0.1030	0.1359	0.1592	3.3000	3.3000	3.3000	3.3000
σ^2^_pheno_	0.0764	0.1448	0.1852	0.2088	3.9638	4.0129	4.0663	3.9311
s.e.	0.0014	0.0031	0.0044	0.0047	0.1486	0.1164	0.1203	0.1037
h^2^	0.03	0.25	0.39	0.31	0.00	0.18	0.39	0.33
s.e.	0.02	0.06	0.07	0.06	0.00	0.06	0.08	0.07
pe^2^	0.01	0.03	0.04	0.01	0.02	0.05	0.06	0.01
s.e.	0.01	0.01	0.01	0.01	0.03	0.02	0.02	0.02
lit^2^	0.21	0.20	0.13	0.15	0.15	0.08	0.03	0.07
s.e.	0.02	0.02	0.02	0.02	0.05	0.03	0.03	0.02

Where σ^2^_sire_ ∼ Sire variance, σ^2^_pe_ ∼ variance of permanent environment due to the dam, σ^2^_lit_ ∼variance due to Litter effects, σ^2^_res_ ∼residual variance, σ^2^_pheno_∼ total phenotypic variance, s.e. ∼ standard error

### 3.4 Genetic trend estimation

**Fig 1:**
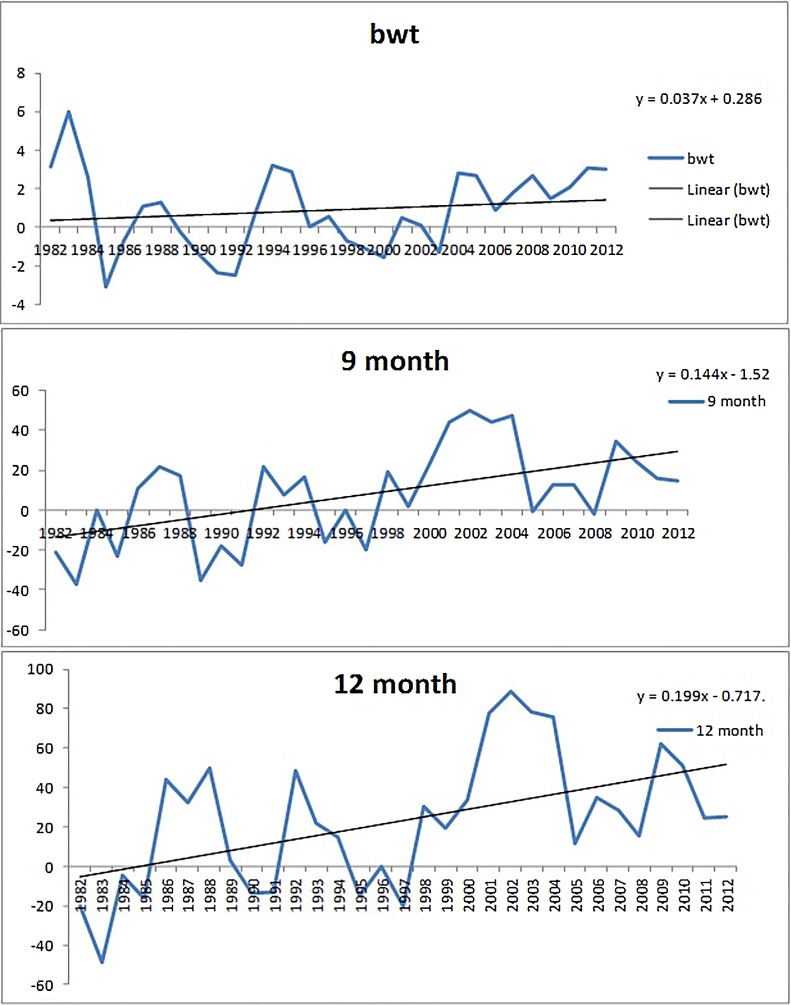
Direct Genetic trend of body weight at birth weight, 9 month and 12 months of age in Jamunapari goats.

**Fig. 2:**
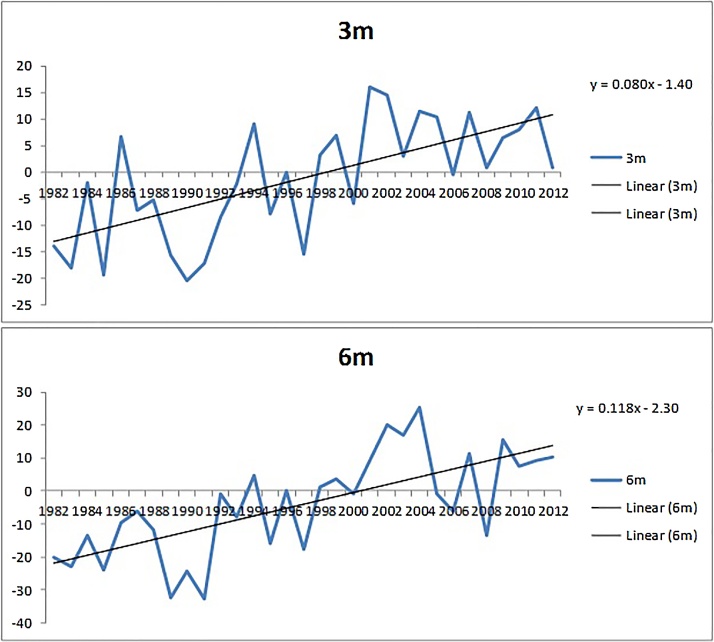
Genetic trend of body weight due to sire effect at 3 month and 6 months of age in Jamunapari goats.

Genetic trends in body weight at various ages are illustrated in Fig 1, Fig. 2. The direct genetic trend at birth, 9 month and 12 months of age are shown in Fig. 1. The genetic trend due to sire effect for 3 month and 6 months of age are presented in Fig. 2. The genetic trend at 9 months of age and 12 months of age was 0.144 kg and 0.199 kg per year, respectively. Similarly, the genetic trend at 3 month and 6 months of age was 0.08 kg and 0.118 kg per year, respectively. The genetic trend at all the ages was very specific and positive during the study period. The genetic trend for birth weight was positive but almost constant in nature.

## 4 Discussion

Although the Jamunapari goats are primarily dairy goats, meat is also important as a secondary product, thus the importance of estimating genetic parameters for growth traits. Maternal and PE are, therefore, important effect to be fitted in the animal model. The estimates of heritability for body traits were low and increased from birth to 3 and 6 months of age and declining thereafter. It may be due to differential gene control at different stages of growth or it may be a sampling bias due to unavailability of same record in the flock due to pre-weaning mortality. In general, higher estimates of heritability were observed when sire was fitted as a random effect as opposed to animal model. The reasons why this is the case is not entirely obvious, as it may be sampling problems with some families losing more kids (higher mortality rate) than others. Similarly, higher estimates of heritability were observed in periods between birth and 6 months of age and the sire model had higher estimates than the animal model. There was no genetic variation for survival at birth with moderate estimates between 9 and 12 months of age. This may be due to high managerial and non-genetic factors. It will be interesting to partition the causes of mortality to understand further the reasons underlying this factor.

The estimates for heritability of body weights at 3–6 months were low to moderate depending on the random effect fitted. The estimates were highest in the sire models and these were higher than those reported in literature for the same age which ranged from 0.06 to 0.17 in different goat breeds (Boujenane and El-Hazzab, 2008; Roy et al., 2008; Hermiz et al., 2009; Ekambaram et al., 2010; Gowane et al., 2011). However, they were within the range (from 0.25 to 0.6) of reported values in the literature for different breeds (Schoeman et al., 1997; Mourad and Anous, 1998; Thiruvenkadan et al., 2009; Faruque et al., 2010; Roy et al., 2011).

Although lower estimates of heritability (0.10–0.12) at 9 months of age were observed in Jamunapari goats, even lower estimates (0.09 ± 0.03) were reported in Sirohi goat at the same age (Gowane et al., 2011). Similarly, the heritability estimates for the body weight at 9 months of age ranged from 0.11 ± 0.04 to 0.30 ± 0.11 in different breeds and locations (Snyman and Olivier, 1996; Roy et al., 2008; Hermiz et al., 2009; Thiruvenkadan et al., 2009; Alade et al., 2010; Ekambaram et al., 2010; Roy et al., 2011). On the other hand, the higher estimates of heritability (>0.33) at 9 months of age were observed in Markhoz goat breed in Iran (Rashidi et al., 2008), Boer goat (Schoeman et al., 1997) and Black Bengal goats (Faruque et al., 2010). The differences in heritability estimates of the present study and those of other workers discussed above may be attributed to the breed difference, size of data set and environmental factors (feeding and management).

The heritability estimates obtained in the present study for weight at 12 months of age were higher than those in Beetal goats (0.07 and 0.09) (Shaiq and Sharif, 1996; Ali and Khan, 2008) at the same age. Other similar heritability estimates for the weight at 12 months of age has been reported in different goat breeds from different countries ranging from 0.07 to 0.11 ± 0.03 (Zhou et al., 2002; Sawalha and Tabbaa, 2004; Gowane et al., 2011). Higher estimates of heritability at 12 months of age ranging from 0.18 ± 0.06 to 0.88 in different breeds (Mukundan and Bhat, 1978; Madeli and Patro, 1984; Jin and Zhang, 1995; Bishop and Russel, 1996; Snyman and Olivier, 1996; Schoeman et al., 1997; Rashidi et al., 2000; Rashidi et al., 2008; Roy et al., 2008; Hermiz et al., 2009; Thiruvenkadan et al., 2009; Ekambaram et al., 2010; Faruque et al., 2010; Roy et al., 2011). The variation in these estimates can be attributed to that the goat breeds vary from pure meat breeds (Boer goat) to dairy breed (Alpine and Saanen breeds) to mixed/dual purpose breeds and some where no selection is practiced.

In general, low estimates of heritability for ADG were reported in literature with estimates of 0.01 for post-weaning daily gain in Angora goat (Gerstmayr, 1988), 0.04 for daily weight gain at six months in Sirohi goat breed (Gowane et al., 2011) and 0.08 for daily weight gain from three to six months of age in Raeini Cashmere goat (Mohammadi et al., 2012). The estimates of heritability for 0–3 month, 3–6 month, 3–12 month, 6–12 month and 9–12 month of age from our current study ranged from 0.04 to 0.47. On the other hand, higher estimate of heritability (0.86) of post weaning body weight were reported in Beetal goats (Shafiq and Sharif, 1996).

The heritability of growth traits was moderately heritable in Jamunapari goats in the present study. Genetic parameter estimates are affected by different population parameter such as number of records, the methods of estimation of heritability, breed, levels of inbreeding, locations and at different ages for trait measurement. The low estimates of heritability for some traits may also suggest that these marginal environments, probably genetic variation is unobservable due to environmental factors. The wide ranges of CV in most of the traits under study indicate a large scope for selection.

The heritability of kid survival was very low and similar estimates have been reported in lamb (Matika et al., 2003; Konstantinov et al., 1994). The low estimates of heritability at weaning age may be due to several reasons such as the susceptibility to bacterial and viral infection is very high during the period due to environmental effect and other management considerations.

The genetic trends obtained for weight at all the ages were positive during the study period. The genetic trend for birth weight was positive but almost constant in nature. Ali and Khan (2008) reported that overall genetic trend for birth weight was static and estimated breeding values ranged from −0.61 to 0.60 kg for bucks. The direct genetic trend at 16-month body weight was 0.11 kg per year, weaning weight increased genetically by 0.06 kg per year while 8-month body weight increased by 0.04 kg per year (Snyman, 2012). The animals in the flock are culled due to health reason only up to 12 months of age. Generally, animals are not culled on the basis of low production till the age of 12 months of age, however the genetic trend is positive and quite high.

## 5 Conclusion

Moderate to high heritability was observed for growth traits at different ages and body weight gain traits during different growth period. Survival of kids at 6, 9 and 12 months of age showed moderate heritability indicating survivability can be improved by selection if the trait is recorded at 6 months of age and above. Genetic trend is positive for all the growth traits indicating selection was successful. However, other traits such as fitness and milk traits will need to be included in a selection index to tailor the breeding programme to different farming settings.

## Conflict of interest

The authors declared no conflict of interest.
