# Supplementary material for: Genetic analysis of growth parameters and survival potential of Jamunapari goats in semiarid tropics
Source: Small Rumin Res. 2018 Aug;165:124–30. doi: 10.1016/j.smallrumres.2018.04.002 (PMC6054051; doi:10.1016/j.smallrumres.2018.04.002)
Supplement: Supplementary file 1 [file mmc1.docx]

Additional file 1

**Table S1** Log–likelihoods obtained for growth traits from birth to 12 months of age under four different models fitting either an animal or sire random effects with the ‘best’ model in bold

| Trait/Model random effects | BWT | M3WT | M6WT | M9WT | M12WT |
| --- | --- | --- | --- | --- | --- |
| **Animal** |  |  |  |  |  |
| a | -3043.2 | -8546.5 | -8151.6 | -7106.2 | -5703.4 |
| a+PE | -2956.8 | -8478.0 | -8127.1 | -7096.6 | -5696.2 |
| a+LIT | -2815.6 | -8416.1 | -8122.5 | -7085.9 | **-5687.5** |
| a+PE+LIT | **-2795.0** | **-8398.2** | **-8112.7** | **-7083.4** | -5685.7 |
|  |  |  |  |  |  |
| **Sire** |  |  |  |  |  |
| a | -3146.3 | -8588.1 | -8185.7 | -7135.0 | -5721.2 |
| a+PE | -2968.3 | -8455.5 | -8123.7 | -7104.4 | -5699.5 |
| a+LIT | -2852.7 | -8419.9 | -8123.7 | -7104.9 | -5698.2 |
| a+PE+LIT | **-2807.6** | **-8379.2** | **-8112.4** | **-7092.5** | **-5690.1** |

Where a ~ additive genetic variance, PE ~ permanent environmental effects due to the dam, LIT ~ litter effects, BWT ~ birth weight; M3WT~ 3 months weight; M6WT~ 6 months weight; M9WT~ 9 months weight; M12WT~ 9 months weight.

**Table S2** Log–likelihoods obtained for average daily gain (ADG) from birth to 12 months of age under four different models fitting either an animal or sire random effects with the ‘best’ model in bold

| Trait | ADG | | | | | | | | | |
| --- | --- | --- | --- | --- | --- | --- | --- | --- | --- | --- |
| Model random effects /months | 0-3 | 3-6 | 3-9 | 3-12 | 0-6 | 6-9 | 6-12 | 0-9 | 9-12 | 0-12 |
| **Animal** |  |  |  |  |  |  |  |  |  |  |
| a | 5702.9 | 4772.5 | 5411.3 | 5548.4 | 6462.8 | 3852.0 | 4607.9 | 6363.1 | 3680.3 | 6199.5 |
| a+PE | 5749.3 | 4775.4 | 5411.6 | 5548.6 | 6479.9 | 3853.1 | 4608.7 | 6369.9 | 3681.9 | 6204.3 |
| a+LIT | 5818.6 | **4792.0** | **5426.4** | **5557.3** | 6486.0 | **3868.9** | **4617.5** | **6380.0** | **3695.5** | **6213.6** |
| a+PE+LIT | **5828.0** | 4792.1 | 5426.4 | 5557.3 | **6492.3** | 3868.9 | 4617.5 | 6381.6 | 3695.5 | 6214.4 |
|  |  |  |  |  |  |  |  |  |  |  |
| **Sire** |  |  |  |  |  |  |  |  |  |  |
| a | 5665.9 | 4771.4 | 5665.9 | 5551.5 | 6433.6 | 3857.5 | 4616.4 | 6336.8 | 3686.3 | 6183.9 |
| a+PE | 5770.2 | 4783.9 | 5412.5 | 5554.6 | 6482.4 | 3860.3 | 4619.4 | 6361.0 | 3690.0 | 6200.6 |
| a+LIT | 5814.7 | 4792.9 | **5426.2** | **5562.2** | 6468.2 | **3875.7** | **4627.2** | 6361.7 | **3702.2** | 6203.9 |
| a+PE+LIT | **5844.6** | **4796.4** | 5426.5 | 5562.4 | **6491.8** | 3875.7 | 4627.4 | **6371.4** | 3702.3 | **6209.6** |

Where a ~ additive genetic variance, PE ~ permanent environmental effects due to the dam, LIT ~ litter effects.
